# Activin A mitigates ferroptosis in cerebral ischemia/reperfusion injury via the PGC-1α/NRF1/TFAM axis

**DOI:** 10.3389/fneur.2026.1842829

**Published:** 2026-06-12

**Authors:** Jiahui Yang, Xiaohua Shi, Ming Ma, Zheng Li, Hongyu Liu, Yang Cui, Zhongxin Xu, Jiaoqi Wang

**Affiliations:** China-Japan Union Hospital, Jilin University, Changchun, China

**Keywords:** Activin A, cerebral ischemia/reperfusion injury, ferroptosis, mitochondrial biogenesis, PGC-1α

## Abstract

**Background:**

Cerebral ischemia/reperfusion (I/R) injury severely limits the efficacy of recanalization therapy for ischemic stroke. Activin A (Act A), a neurotrophic cytokine, shows protective potential, but its mechanisms related to mitochondrial biogenesis and ferroptosis regulation remain unclear.

**Methods:**

*In vivo*, adult male Wistar rats (12/group) underwent 2 h middle cerebral artery occlusion (MCAO) and 24 h reperfusion. Act A (7.5 μg/kg) or vehicle was administered intracerebroventricularly pre-ischemia. TTC, TEM, and IHC were used to analyze cerebral I/R injury and PGC-1α expression. *In vitro*, HT22 cells exposed to oxygen–glucose deprivation/reoxygenation (OGD/R, 8 h/24 h) were treated with Act A (100 ng/mL). Ferroptosis markers, mitochondrial function and signaling pathways were assessed via qPCR, western blot, flow cytometry, laser confocal and ChIP.

**Results:**

*In vivo*, Act A significantly reduced cerebral infarct volume versus vehicle (***p < 0.001), decreased MDA levels (***p < 0.001), and increased PGC-1α expression (***p < 0.001) along with mtDNA copy number (*p < 0.05). *In vitro*, Act A rescued OGD/R-induced ferroptosis, suppressing lipid ROS (**p < 0.01) and Fe^2+^ accumulation (*p < 0.05). It activated the PGC-1α/NRF1/TFAM axis (*p < 0.05) and enhanced mitochondrial biogenesis. Mechanistically, Act A promoted PGC-1α transcription via Smad3 binding to its promoter (*p < 0.05) and enhanced PGC-1α activity through p38-MAPK phosphorylation (***p < 0.001). Silencing PGC-1α abolished Act A’s neuroprotection effects.

**Conclusion:**

Act A mitigates cerebral I/R injury by dual activation of PGC-1α through Smad3 and p38-MAPK pathways, enhancing mitochondrial biogenesis and inhibiting neuronal ferroptosis. This highlights Act A as a therapeutic candidate for ischemic stroke.

## 1 Introduction

Ischemic stroke poses a major public health challenge due to its significant morbidity and mortality, with projections indicating rising incidence, prevalence, and mortality rates over the next two decades (1). Early vascular recanalization remains the most effective intervention in the acute phase. Advances in cerebral perfusion imaging have extended the therapeutic window for intravenous thrombolysis (IVT) and endovascular thrombectomy (EVT) to 24 h (2–4). However, reperfusion therapies may trigger cerebral ischemia/reperfusion (I/R) injury, which may lead to secondary neurological deterioration and cerebral edema, substantially compromising the outcomes of patients. The pathogenesis of cerebral I/R injury involves complex mechanisms, including oxidative stress, mitochondrial dysfunction, calcium overload, and inflammatory cascades. Therefore, further elucidation of these molecular pathways and identification of novel therapeutic targets are critical for improving clinical prognosis (5).

Oxidative stress plays a pivotal role in cerebral I/R injury, primarily driven by neuronal mitochondrial damage and subsequent reactive oxygen species (ROS) burst (6). Ferroptosis is an iron-dependent form of regulated cell death that is characterized by uncontrolled ROS accumulation and lipid peroxidation and represents a principal modality of cell death in cerebral I/R injury (7). Maintaining mitochondrial homeostasis represents a critical defense strategy against ferroptosis, encompassing mitochondrial biogenesis, fission-fusion dynamics, and mitophagy. These coordinated processes are indispensable for cellular energy production, maintenance of mitochondrial architecture and function, and selective removal of damaged organelles (8, 9).

Mitochondria possess an autonomous self-replicating genome known as mitochondrial DNA (mtDNA). mtDNA encodes 13 essential subunits of the electron transport chain (ETC), which are critical for oxidative phosphorylation (10). Mitochondrial biogenesis increases mitochondrial mass and number in response to elevated cellular energy demands through tightly coordinated transcription and translation of both nuclear and mitochondrial genomes. Peroxisome proliferator-activated receptor gamma coactivator 1-*α* (PGC-1α) serves as the master regulator of this process, recruiting nuclear transcription factors, including nuclear respiratory factor 1 (NRF1). NRF1 activates mitochondrial transcription factor A (TFAM), the primary regulator of mtDNA replication and transcription (11). Notably, studies have revealed that mtDNA depletion syndromes increase the susceptibility to iron overload-induced ferroptosis, suggesting that mtDNA-supported mitochondrial homeostasis plays a critical role in regulating ferroptosis (12). Previous studies have shown that PGC-1α activation can enhance mitochondrial biogenesis and effectively suppress neuronal ferroptosis (13).

Act A, a member of the transforming growth factor-*β* (TGF-β) superfamily, is a homodimeric protein composed of two βA subunits linked by disulfide bonds. Like other ligands of the TGF-β family, Act A initiates signaling through sequential engagement of serine/threonine kinase receptors, activating the downstream Smad transcription factor. Phosphorylated Smad2 and Smad3 form a heteromeric complex with Smad4, and the activated Smad complex translocates to the nucleus where it interacts with transcriptional cofactors and regulates the expression of target genes (14). Additionally, Act A activates non-canonical signaling pathways, particularly p38-MAPK and ERK1/2, thereby modulating cellular proliferation and differentiation (15). As a secretory cytokine widely expressed in the central nervous, circulatory, respiratory, and reproductive systems, Act A regulates fundamental biological processes, including cell proliferation, differentiation, and metabolism (16). In the central nervous system, Act A serves as a pivotal neuromodulator that induces specific neuronal phenotypes, enhances synaptic plasticity in hippocampal neurons, functions as a dynamic biomarker of neuronal activity, and acts as a neurotrophic factor (17).

The Act A/Smad signaling axis exhibits significant crosstalk with mitochondrial quality control systems governing the homeostasis of cellular organelles. The Act A/Smad3 axis modulates inflammatory responses by regulating mitochondrial ATP production in macrophages. Smad3-knockout macrophages exhibit significantly reduced mitochondrial mass and diminished ATP levels, indicating that Act A controls the abundance of mitochondria and cellular bioenergetics through canonical Smad signaling pathways (18). Similarly, it has been observed that the mDia2-MIRO1 axis controls mitochondrial positioning and function in fibroblasts. Notably, a Smad-binding element resides upstream of the *MIRO1* transcriptional start site, and Act A promotes Smad2/3 binding to enhance the expression of *MIRO1* and subsequently regulate mitochondrial function (19). The *INHBA* gene encodes the βA subunit of Act A. Researchers generated homozygous InhbaBK/BK and heterozygous InhbaBK/− mice by inserting *INHBB* gene sequences into the genomic domain of *INHBA*. Analysis of the expression of genes linked to energy metabolism and mitochondrial function revealed significantly elevated mRNA levels of *PGC-1α*, *NRF1*, and *NRF2* in the liver, skeletal muscle, and brown adipose tissues of InhbaBK/− heterozygotes compared to InhbaBK/BK homozygotes. These findings suggest that the expression of the βA subunit potentially modulates transcriptional regulation of genes associated with mitochondrial biogenesis (20). Furthermore, the p38-MAPK phosphorylation cascade plays a critical role in PGC-1α activation, while p38-MAPK phosphorylation represents one of the key non-canonical intracellular signaling pathways downstream of Act A (21).

This study elucidated the molecular mechanism through which Act A regulates mitochondrial homeostasis and confers neuroprotection against cerebral I/R injury. Using complementary MCAO/R rat models and OGD/R cellular systems, we found that ferroptosis constitutes a pivotal mode of regulated neuronal death after I/R. Act A orchestrates the synergistic activation of PGC-1α through dual Smad3/p38-MAPK signaling pathways, enhancing mitochondrial biogenesis to suppress neuronal ferroptosis and ameliorate cerebral I/R injury. Our findings advanced our mechanistic understanding of the role of the mitochondrial homeostasis-ferroptosis axis in ischemic stroke, establishing a therapeutic rationale for targeting the Act A-PGC-1α cascade in cerebral I/R injury.

## 2 Materials and methods

### 2.1 Animal

Adult male Wistar rats (180–220 g body weight) were maintained in standard laboratory housing at 25 ± 2 °C and 50 ± 5% relative humidity, adhering to a 12-h light/dark schedule. Food and water were available ad libitum throughout the study. All experimental procedures strictly followed the guidelines of the Institutional Animal Care and Use Committee (IACUC) and received prior approval (SY: 2025–12-006) from the School of Public Health, Jilin University.

### 2.2 The treatment of Act A

Prior to surgery, the animals underwent a 12 h fasting period. Drug administration occurred 1 h preoperatively. Act A (HEK293, MCE, USA) was prepared by dissolving 50 μg in 50 μL of 0.1% BSA, resulting in a stock solution of 1 μg/μL. Act A was administered via intracerebroventricular microinjection at a dose of 7.5 μg/kg body weight. Stereotaxic coordinates for injection into the right lateral ventricle, relative to the bregma, were determined as follows: X-axis +1.40 mm, Y-axis −0.36 mm, Z-axis −3.90 mm. The total injection volume did not exceed 2 μL.

### 2.3 Middle cerebral artery occlusion/reperfusion (MCAO/R) model

The rats were anesthetized via intraperitoneal injection of pentobarbital sodium (40 mg/kg) and positioned supine on a surgical platform. After hair removal and skin disinfection, a midline longitudinal incision was made in the neck. The carotid sheath was bluntly dissected to expose and isolate the right common carotid artery (CCA), external carotid artery (ECA), and internal carotid artery (ICA), with care taken to avoid manipulation or stimulation of the vagus nerve and carotid sinus. The proximal ECA and CCA were temporarily occluded using microvascular clips. Blood flow through the ICA was temporarily halted with a sterile ligature. A small incision was made in the ECA using micro-scissors, and a monofilament suture was advanced gently through the ECA into the ICA until mild resistance indicated occlusion of the MCA origin. After 2 h of occlusion, the filament was slowly withdrawn to restore cerebral blood flow, which was maintained for 24 h. Sham-operated animals underwent identical surgical procedures, including vessel isolation and incision, but without filament insertion.

Postoperatively, rats were placed on a heating pad to maintain body temperature at 37 ± 0.5 °C. Following 24 h of reperfusion, neurological deficit scores were assessed. Rats were then euthanized, and brain tissues were harvested for subsequent analysis.

According to the blood supply region of the MCA and referencing the method of Ashwal et al. (22), cut 6 mm thick coronal sections at 3 mm and 9 mm from the frontal pole of the brain. At 2 mm lateral to the sagittal suture in the right cerebral hemisphere, remove the left side and intermediate structures supplied by the anterior cerebral artery. Make an oblique cut at 30° to the sagittal plane 2 mm lateral to the sagittal cut in the remaining brain section, with the lateral cortex being the ischemic core and the medial part being the ischemic penumbra. Collect cortical tissue from the ischemic penumbra for biochemical tests.

### 2.4 Neurological functional score

Neurological deficits were evaluated in all rats following 24 h of reperfusion using the Bederson scoring system. Assessment was based on a 4-point scale:

0: No observable neurological deficits.

1: Failure to fully extend the contralateral forelimb.

2: Circling to the contralateral side.

3: Falling to the contralateral side.

4: Absence of spontaneous motor activity accompanied by a depressed level of consciousness.

### 2.5 Measurement of cerebral infarction volume

Following 24 h of reperfusion, rats were euthanized, and whole brains were immediately harvested. Place the brain tissue in a − 20 °C freezer for 30 min. The brain was sectioned coronally into 5 serial slices of 2 mm thickness with a sharp blade. Sections were incubated for 30 min in 37 °C pre-warmed 0.5% TTC solution (G1017, Solarbio, China). Healthy brain tissue stained red, whereas infarcted tissue appeared white. Transfer the brain sections into 4% paraformaldehyde solution and fix for 20 min. Digital images of the stained sections were acquired, and quantitative analysis was performed using Image J software. Infarct volume was expressed as a percentage of total brain volume: Infarct % = infarct volume / total brain volume × 100%.

### 2.6 HE staining

After sacrifice, rat brain tissues were fixed overnight in 4% paraformaldehyde (P1110, Solarbio, China), rinsed with tap water, and dehydrated through a graded ethanol series. Following paraffin embedding, tissue blocks were sectioned at 5 μm thickness using a microtome. Sections were stained with HE staining (G1120, Solarbio, China) according to standard protocols. Stained slides were analyzed and observed under a scanner.

### 2.7 Transmission electron microscopy (TEM)

Fresh brain tissues were rapidly immersed in fixative for electron microscopy and fixed at 4 °C for 2 h, followed by fixation in 1% osmic acid ·0.1 mmol/L phosphate buffer at room temperature for 2 h. Tissues were then dehydrated through a graded ethanol-acetone series. After embedding, ultrathin sections were cut using an ultramicrotome, stained, and allowed to dry. Sections were examined under a transmission electron microscope (JEM1400PLUS, JEOL, Japan).

### 2.8 MDA measurement

Fresh peri-infarct cortex tissues or HT22 cell were complete lysed in lysis buffer. Protein concentrations were determined using a BCA protein assay kit (P0010, Beyotime, China). Using an MDA assay kit (BC0025, Solarbio, China), working solution was prepared and mixed thoroughly with sample supernatant according to the manufacturer’s instructions. The mixture was incubated at 100 °C in a heating block for 60 min. After cooling and centrifugation, absorbance was measured at 532 nm and 600 nm using a microplate reader. MDA content (nmol/mg prot) = 53.763 × ΔA/Cpr.

### 2.9 Oxygen–glucose deprivation/reoxygenation (OGD/R) cell model

Following cell counting and plating, cells were incubated overnight in a CO_2_ incubator. Upon adhesion, cultures were rinsed with PBS to remove complete medium and switched to glucose-free medium. Cells were then placed in a tri-gas incubator (37 °C, 1% O_2_, CO_2_ and 94% N_2_) for 8 h. The glucose-free medium was subsequently replaced with high-glucose medium supplemented with 1% fetal bovine serum, and transferred to a normoxic CO_2_ incubator for 24 h reoxygenation. Cells were harvested for subsequent experiments after reoxygenation. Cells were treated with 100 ng/mL Act A during OGD/R.

### 2.10 CCK-8 measurement

After trypsinization and resuspension, cells were counted and diluted to a density of 5,000 cells/100 μL medium. The cell suspension was thoroughly mixed and seeded into 96-well plates, with triplicate wells per group. Following overnight incubation in a CO_2_ incubator, adherent cells underwent OGD and Act A treatment. At designated time points, culture medium was replaced with 100 μL DMEM containing 10 μL pre-mixed CCK-8 reagent (BA00208, Bioss, China). After 1–2 h of light-protected incubation at 37 °C, absorbance at 450 nm was measured using a microplate reader. The formula specified in the kit protocol was applied to calculate cell viability.

### 2.11 Immunohistochemistry

Deparaffinized brain tissue sections underwent antigen retrieval in EDTA buffer (G1207, Servicebio, China). To block non-specific binding, sections were incubated with 3% BSA (G5001, Servicebio, China) for 30 min at room temperature. After blocking, sections were incubated overnight at 4 °C with primary anti-PGC-1α antibody (ab188102, Abcam, USA, 1:200). The following day, sections were incubated with goat anti-rabbit secondary antibody (GB23303, Servicebio, China, 1:500) for 1 h at room temperature. DAB chromogenic substrate (G1211, Servicebio, China) was applied with microscopic monitoring until tan-brown positive staining developed. Sections were then counterstained with hematoxylin, dehydrated, mounted, and imaged microscopically.

### 2.12 qRT-PCR

The CDS sequence of the target gene was identified on the NCBI website. Primers for the target gene were designed using the Sangon Biotech online primer design platform, with primer sequences listed as follows: ND1 (sense, 5′- CGAGCCGTAGCCCAAACAATTTC-3′, antisense, 5′- CTATGGGTCAGGCTGGCAGAAG-3′), PGC-1α (sense, 5′- TATGGAGTGACATAGAGTGTGCT-3′, antisense, 5′- GTCGCTACACCACTTCAATCC-3′), NRF1 (sense, 5′- GGCAACAGTAGCCACATTGGCT-3′, antisense, 5′- GTCTGGATGGTCATTTCACCGC-3′) and TFAM (sense, 5′-ATTCCGAGTGTTTTTCCAGCA-3′, antisense, 5′- TCTGAAAGTTTTGCATCTGGGT-3′).

Total DNA was extracted from tissues and cells using the Genomic DNA Purification Kit (B0007, EZBioscience, USA). DNA concentration was quantified, and all samples were diluted equivalently. qPCR was performed using SYBR Green qPCR Mix (AH0104-B, RK21204; Shandong Sparkjade Biotechnology Co., Ltd., China). DNA templates were added to qPCR reaction systems, with each DNA sample subjected to amplification using two distinct primer pairs (*mt-ND1* and *β-actin*) under specified thermal cycling conditions. Relative mtDNA content was calculated using the formula: 2^(-ΔΔCt).

Total RNA was isolated from cells using an RNA Extraction Kit (AC0202; Shandong Sparkjade Biotechnology Co., Ltd., China). RNA concentration was measured with a microplate reader. Reverse transcription was performed using the ACScript RT PreMix Kit (RK20429; Abclonal, China), with reaction components added to nuclease-free tubes according to the manufacturer’s protocol. RNA was reverse-transcribed into cDNA in a thermal cycler. cDNA samples were uniformly diluted and added to qPCR reaction systems. Amplification was conducted on a fluorescence quantitative PCR system (C1000 Thermal Cycler, Bio-Rad, USA) with predetermined temperature profiles and cycle numbers. Gene expression levels were normalized to the reference gene *β-actin*.

### 2.13 Western blot

Extract total protein from brain tissues or cells, add RIPA lysis buffer containing 1 × protease inhibitor and phosphatase inhibitor cocktail according to tissue weight or cell count. Thoroughly lyse tissues or cells using a tissue homogenizer and sonication, with all procedures performed on ice. Centrifuge and collect the supernatant, then determine protein concentration using a BCA protein assay kit (P0010, Beyotime, China). Prepare 7.5 –15% SDS-PAGE (EC0023, Shandong Sparkjade Biotechnology Co., Ltd., China) and perform electrophoresis. Then transfer proteins onto PVDF membranes (IPFL00005, Millipore, USA) via electrotransfer and block with 5% skim milk. Place the PVDF membrane into an appropriately sized sealing bag, dilute primary antibodies with antibody dilution buffer, and incubate overnight at 4 °C. Primary antibodies included anti-PGC-1α (ab188102, Abcam, USA, 1:1000), anti-NRF1 (ab175932, Abcam, USA, 1:1000), anti-TFAM (ab307302, Abcam, USA, 1:1000), anti-Smad2/3 (8,685 T, CST, USA, 1:1000), anti-Phospho-Smad2/3 (8,828 T, CST, USA, 1:1000), anti-P38 MAPK (8,690 T, CST, USA, 1:1000), anti-Phospho-P38 MAPK (4,511 T, CST, USA, 1:1000), anti-ACSL4 (ab155282, Abcam, USA, 1:1000) and anti-GPX4 (ab125006, Abcam, USA, 1:1000). The next day, transfer the PVDF membrane to a light-proof box, incubate with fluorescence-labeled secondary antibody (5151S, Cell Signaling Technology, USA, 1:10,000) diluted in TBS at room temperature for 1 h. Place the PVDF membrane on the Odyssey dual-color infrared laser imaging system (LICOR Odyssey, USA), adjust appropriate parameters, scan the membrane, and analyze images using ImageJ software.

### 2.14 Flow cytometry detect lipid ROS, mitochondrial content, and mitochondrial membrane potential (ΔΨm)

Following trypsinization, harvested cells were resuspended in serum-free DMEM. Fluorescent probes were prepared at specified concentrations: the stock solution of the Lipid Peroxidation Probe 581/591 C11 (L267, DOJINDO, Japan) was diluted 1,000-fold to assess cellular lipid ROS levels; MitoTracker Green FM (HY135056, MCE, USA) diluted to 100 nmol/L for assessing mitochondrial mass; and JC-1 (HY15534, MCE, USA) diluted to 2 μmol/L for detecting mitochondrial membrane potential (ΔΨm). Cell suspensions were incubated with fluorescent dyes according to the manufacturer’s protocols and subsequently analyzed by flow cytometry (Becton CYTOMINCS FC500, USA), with acquired data analyzed by FlowJo v10.8.1 software.

### 2.15 Laser confocal detect lipid ROS and Fe^2+^

Cells in optimal growth condition were trypsinized, counted, and seeded into confocal dishes at a density of 1 × 10^5^ cells per dish. Following gentle agitation, dishes were transferred to a CO₂ incubator for a minimum of 24 h to achieve complete adhesion before OGD/R and Act A treatments. For cellular Fe^2+^ quantification, 200 μL of a 1 μmol/L solution of Ferro Orange fluorescent probe (F374, DOJINDO, Japan) was added to each confocal dish. After light-protected incubation at 37 °C for 5 min, the staining solution was aspirated, and cells were washed twice with PBS. Fluorescence was imaged under 561 nm excitation using a laser scanning confocal microscope (FV1000, Olympus, Japan). For lipid ROS assessment, 200 μL of of a 1,000-fold diluted solution of Lipid Peroxidation Probe 581/591 C11 (L267, DOJINDO, Japan) was added to each dish. Following 30 min of light-protected incubation at 37 °C, the staining solution was removed, and cells underwent two PBS washes. Dual-excitation fluorescence was detected at 488 nm and 561 nm via confocal microscopy.

### 2.16 ATP measurement

HT22 cells were seeded in white-walled 96-well plates at a density of 5,000 cells per well and cultured overnight in a CO_2_ incubator. Subsequently, cells underwent OGD/R treatment, while control groups were maintained under standard CO_2_ incubator conditions. Cellular ATP levels were quantified using an ATP detection kit (CK18, DOJINDO, Japan) by adding 100 μL of working solution to each well, followed by orbital shaking for 2 min. The plate was then dark-adapted in the microplate reader chamber at a controlled temperature of 25 °C for 10 min to stabilize luminescent signals. Relative light units were measured, with luminescence intensity directly proportional to intracellular ATP concentration.

### 2.17 Si-RNA transfection

Both control siRNA and PGC-1α-targeting siRNA were diluted in Opti-MEM medium, where the control siRNA sequences comprised sense 5′-UUCUCCGAACGUGUCACGUTT-3′ and antisense 5′-ACGUGACACGUUCGGAGAATT-3′, while the *PGC-1α* siRNA sequences included sense 5′-GCCCUAUUCAUUGUUCGAUTT-3′ and antisense 5′-AUCGAACAAUGAAUAGGGCCTT-3′; subsequently, a 100 μL mixture of siRNA-HiTransfer (TF0100, IBSBIO, China) was introduced to cells cultured in 6-well plates at approximately 60% confluency, followed by incubation periods of 24 h, 48 h, and 72 h prior to cell harvesting for quantitative assessment of *PGC-1α* silencing efficiency via qRT-PCR and Western blotting.

### 2.18 Chromatin immunoprecipitation (ChIP)

Prepare approximately 10^7 cells, add PBS containing 1% formaldehyde (47,608, Sigma, USA) for fixation and 10 × glycine solution (50,046, Sigma, USA) to quench fixation, then collect cell pellet. Add ice-cold cell swelling buffer, lyse on ice for 10 min, then centrifuge to collect pellet. Resuspend pellet in ice-cold SDS lysis buffer, lyse on ice for 10 min, sonicate cells and collect supernatant after centrifugation. Using the BeyoChIPTM ChIP Assay Kit (P2083S, Beyotime, China), prepare ice-cold ChIP dilution buffer dilute supernatant to 2 mL. Reserve 100 μL as Input sample, divide remaining liquid into negative control group and IP group, add IgG antibody (3,900 T, CST, USA, 1:100) and p-Smad3 antibody (9,520 T, CST, USA, 1:100) respectively, incubate with rotation overnight at 4 °C. Add Protein A/G Magnetic Beads/Salmon Sperm DNA to each group, incubate with rotation at 4 °C for 1 h. Wash the formed bead-antibody-protein-DNA complexes sequentially with: low Salt, high Salt, LiCl immune complex wash buffer once, and TE buffer twice. Add 200 μL elution buffer to negative control and IP groups respectively, incubate in 65 °C water bath for 15 min with inversion every 2 min, collect supernatant after magnetic separation. Add NaCl and proteinase K to all three groups, incubate overnight at 65 °C in a thermomixer. Purify using MinElute PCR Purification Kit (28,004, Qiagen, Germany) and reserve eluates. Retrieve PGC-1α promoter sequence from NCBI database, design PCR primers using Primer Premier 6.0 and Beacon Designer 7.8 software, synthesize three primer pairs: PGC-1α-1 (sense, 5′- CCCACTGGCCTCTAGTGATA −3′, antisense, 5′- GGAATCCATGTCCTTCCCAATAG -3′), PGC-1α-2 (sense, 5′- CCTCACCCCATCAGTTACCTA −3′, antisense, 5′- CGTGCCCTGTGTTATTTTGCAGA -3′), PGC-1α-3 (sense, 5′- GGCGTAGCTGCTAGCTTTGT −3′, antisense, 5′- GCCGATGGTATCATCAAGGATGGAA −3′). Perform qPCR detection on all three sample groups.

### 2.19 Statistical analysis

All experiments included at least three independent biological replicates, with TTC staining comprising six independent replicates per group. Data were statistically analyzed using GraphPad Prism 10.1.2 software. Comparisons between two groups were performed using unpaired t-test. Comparisons among three or more groups were assessed by one-way ANOVA. For non-normally distributed data, comparisons between two independent groups used Mann–Whitney U test, while comparisons among three or more groups used Kruskal-Wallis H test. *p* < 0.05 was considered statistically significant. *p* < 0.01, *p* < 0.001, and *p* < 0.0001 were considered highly statistically significant.

## 3 Results

### 3.1 Act A ameliorated cerebral I/R injury and promoted PGC-1α expression in rats undergoing MCAO/R

**Figure 1:**
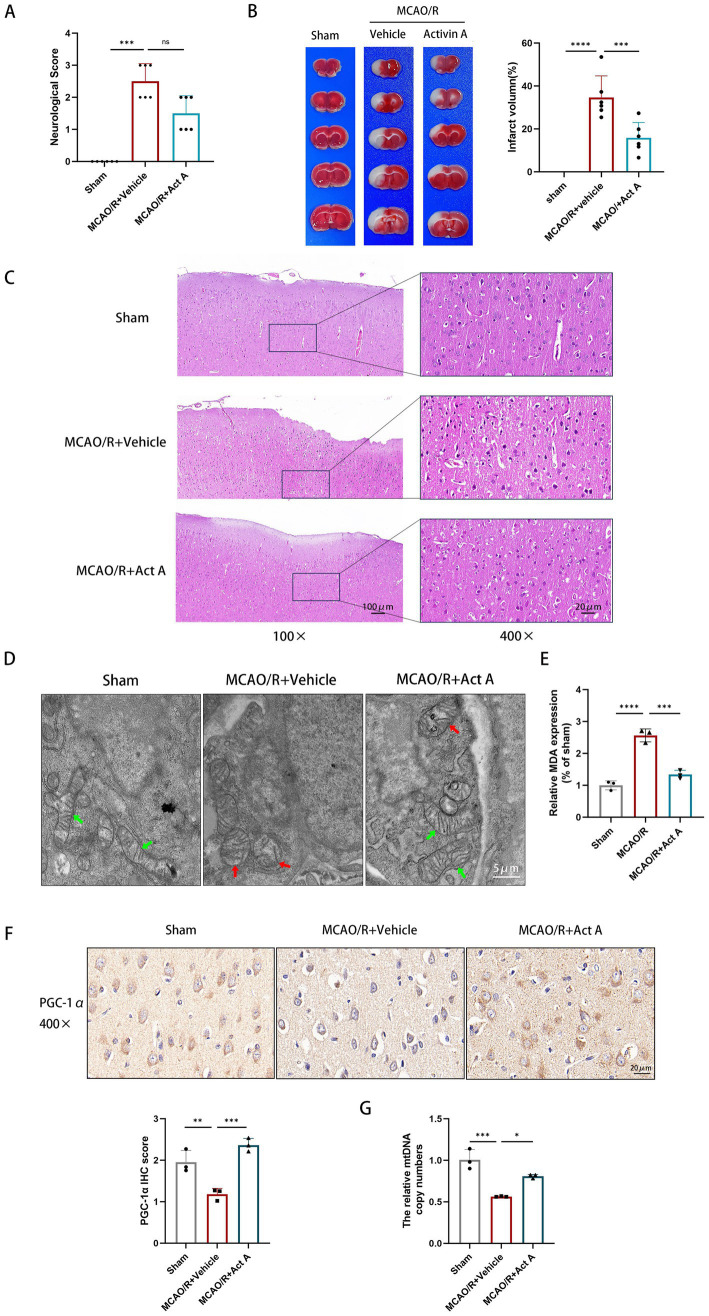
Act A ameliorates tissue damage and neuronal ferroptosis while promoting PGC-1α expression and mitochondrial biogenesis in the MCAO/R model. **(A)** Neurological deficits assessed by Bederson scoring system (***p < 0.001, *n* = 6/group). **(B)** Representative TTC-stained sections and quantification of cerebral infarct volume (***p < 0.001; ****p < 0.0001, *n* = 6/group). **(C)** Histopathological evaluation by HE staining in the peri-ischemic cortex (magnification, 100 × and 400×; scale bars, 100 μm and 20 μm). **(D)** Transmission electron microscopy showing mitochondrial ultrastructure (red arrows: ferroptotic mitochondria; green arrows: normal mitochondria; magnification, 30,000×; scale bar, 0.5 μm). **(E)** Cortical MDA levels in peri-ischemic tissues (***p < 0.001; ****p < 0.0001, *n* = 3/group). **(F)** Immunohistochemical analysis of PGC-1α expression in the cortex (**p < 0.01; ***p < 0.001, *n* = 3/group; scale bar, 20 μm). **(G)** Relative mtDNA content (ND1/β-actin ratio) measured by qPCR in peri-ischemic tissues (*p < 0.05; ***p < 0.001, *n* = 3/group). Data are represen ted as mean ± SEM.

To evaluate the neuroprotective effects of Act A on cerebral I/R injury, this study divided rats into the sham, MCAO/R+vehicle, and MCAO/R+Act A groups. Neurological function was assessed 24 h after reperfusion using the Bederson scoring system. Rats in the MCAO/R+vehicle group exhibited typical neurological deficits, including circling and falling toward the paralyzed side. Although paralysis was milder in the MCAO/R+Act A group, the Bederson scores of this group did not show a significant difference compared to the MCAO/R+vehicle group (Figure 1A). In TTC staining, the Act A-treated group showed a significant reduction in infarct percentage compared to the MCAO/R+vehicle group (Figure 1B). HE staining was conducted to observe histopathological changes in the peri-ischemic cortical region across the groups. The MCAO/R+vehicle group exhibited characteristic features of ischemic injury, including deranged and sparse neuronal alignment, pyknotic nuclei, and significant interstitial edema. In contrast, the Act A-treated group revealed more organized neuronal arrangement, preserved nuclear structures, and milder interstitial edema compared to the model group (Figure 1C). These findings suggest that treatment with Act A may alleviate histopathological damage in rats subjected to MCAO/R.

TEM was employed to investigate the ultrastructural changes of neuronal mitochondria in the peri-ischemic cortical region. The results revealed that neurons in the MCAO/R+vehicle group exhibited mitochondrial alterations characteristic of ferroptosis, including reduced mitochondrial volume with increased membrane density (red arrows). The MCAO/R+Act A group showed a reduced number of ferroptotic mitochondria (Figure 1D). The MCAO/R+vehicle group indicated significantly elevated MDA levels compared to the sham group. Treatment with Act A significantly reduced MDA concentrations in rats undergoing MCAO/R (Figure 1E). These results suggest that treatment with Act A can mitigate neuronal ferroptosis and inhibit lipid peroxidation in rats undergoing MCAO/R. To investigate the effects of Act A on mitochondrial biogenesis in cerebral I/R injury, we measured PGC-1α expression in the peri-ischemic cortex using immunohistochemical staining. The MCAO/R+vehicle group exhibited significantly reduced expression levels of PGC-1α compared to the sham group. Conversely, treatment with Act A markedly increased PGC-1α levels compared to the MCAO/R+vehicle group (Figure 1F). Furthermore, qRT-PCR of relative mtDNA copy number revealed a significant decrease in the ND1/*β*-actin ratio in the MCAO/R+vehicle group compared to the sham group, indicating impaired mtDNA synthesis following I/R injury. Treatment with Act A significantly elevated the ND1/β-actin ratio in rats subjected to MCAO/R (Figure 1G). Collectively, these findings suggest that Act A may mitigate neuronal ferroptosis in rats undergoing MCAO/R and may promote mitochondrial biogenesis by upregulating PGC-1α expression.

### 3.2 Activin A mitigated neuronal ferroptosis in an *in vitro* model of cerebral I/R injury

**Figure 2:**
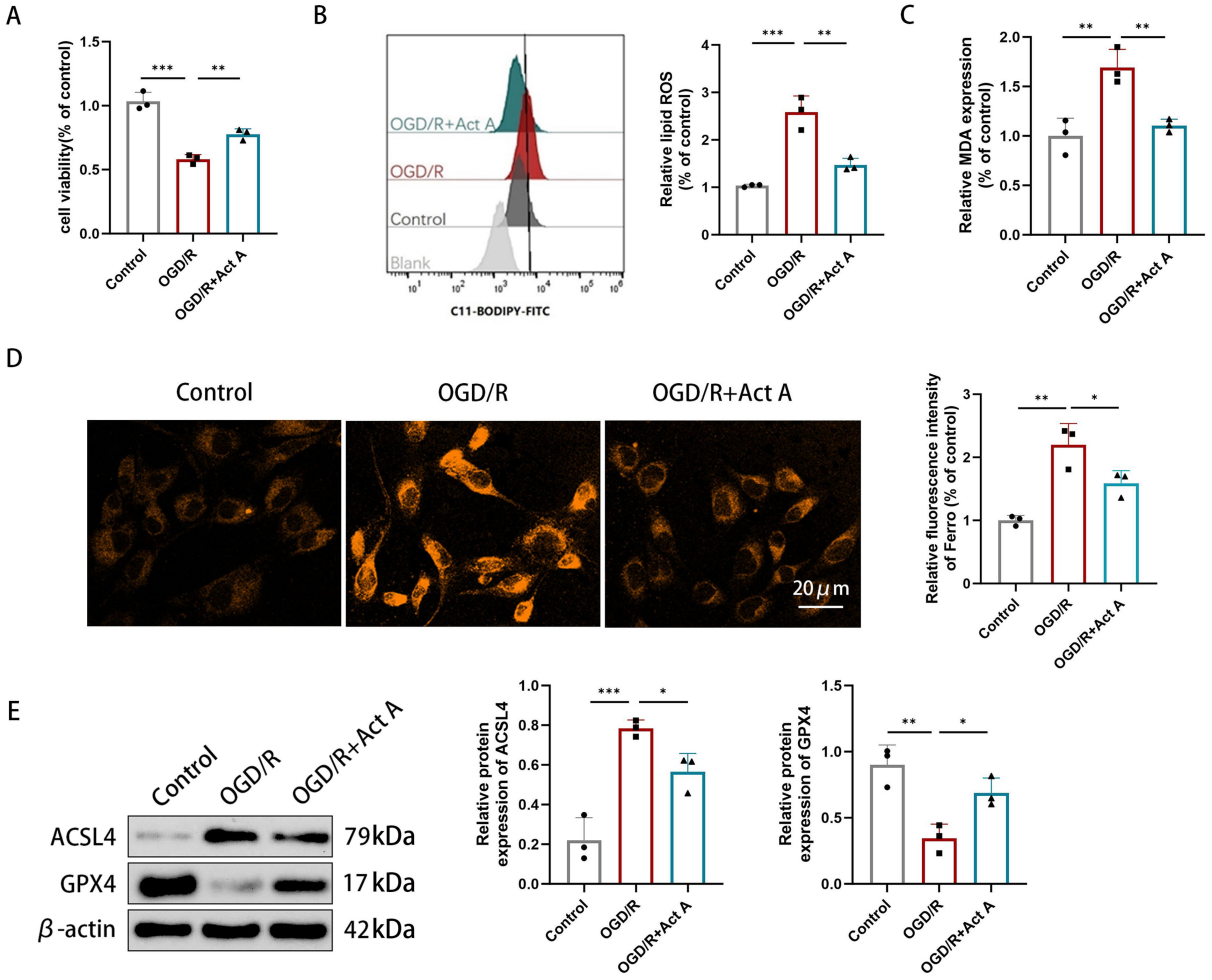
Act A mitigates neuronal ferroptosis in OGD/R-induced injury. **(A)** Cell viability assessed by CCK-8 assay (**p < 0.01; ***p < 0.001, *n* = 3/group). **(B)** Lipid ROS levels measured by flow cytometry (**p < 0.01; ***p < 0.001, *n* = 3/group). **(C)** Cellular MDA content quantification (**p < 0.01, *n* = 3/group). **(D)** Intracellular Fe^2+^ levels detected by laser confocal microscopy (scale bar, 20 μm, *p < 0.05; **p < 0.01, *n* = 3/group). **(E)** Western blot analysis of ferroptosis markers ACSL4 and GPX4 (*p < 0.05; **p < 0.01; ***p < 0.001, *n* = 3/group). Data are represen ted as mean ± SEM.

To delineate the neuroprotective effects of Act A in an in vitro model of cerebral I/R injury, HT22 cells were divided into three groups, including control, OGD/R, and OGD/R+Act A. Cell viability was assessed by CCK-8 assay and revealed that treatment with Act A significantly improved survival rates compared to the OGD/R group (Figure 2A). Since lipid peroxidation constitutes a core event in ferroptosis, we measured lipid ROS levels using C11-BODIPY™ fluorescent probe coupled with flow cytometry. The OGD/R group exhibited significantly elevated levels of lipid ROS compared to controls, whereas treatment with Act A substantially attenuated lipid ROS accumulation in OGD/R cells (Figure 2B). Consistently, MDA quantification demonstrated significantly reduced lipid peroxidation in Act A-treated cells compared to the OGD/R group (Figure 2C). Laser confocal microscopy also revealed significantly increased Fe^2+^ concentrations in OGD/R cells compared to control cells, which was effectively reversed after treatment with Act A (Figure 2D). Western blotting of ferroptosis marker proteins showed that treatment with Act A significantly upregulated GPX4 expression and downregulated ACSL4 levels in OGD/R cells (Figure 2E). Such alterations in ferroptosis regulators are mechanistically linked to inhibition of lipid peroxidation. Collectively, these results suggest that Act A exerts neuroprotective effects against OGD/R-induced injury primarily by suppressing ferroptotic lipid peroxidation.

### 3.3 Activin A promoted mitochondrial biogenesis in OGD/R cells by activating the PGC-1α/NRF1/TFAM pathway

**Figure 3:**
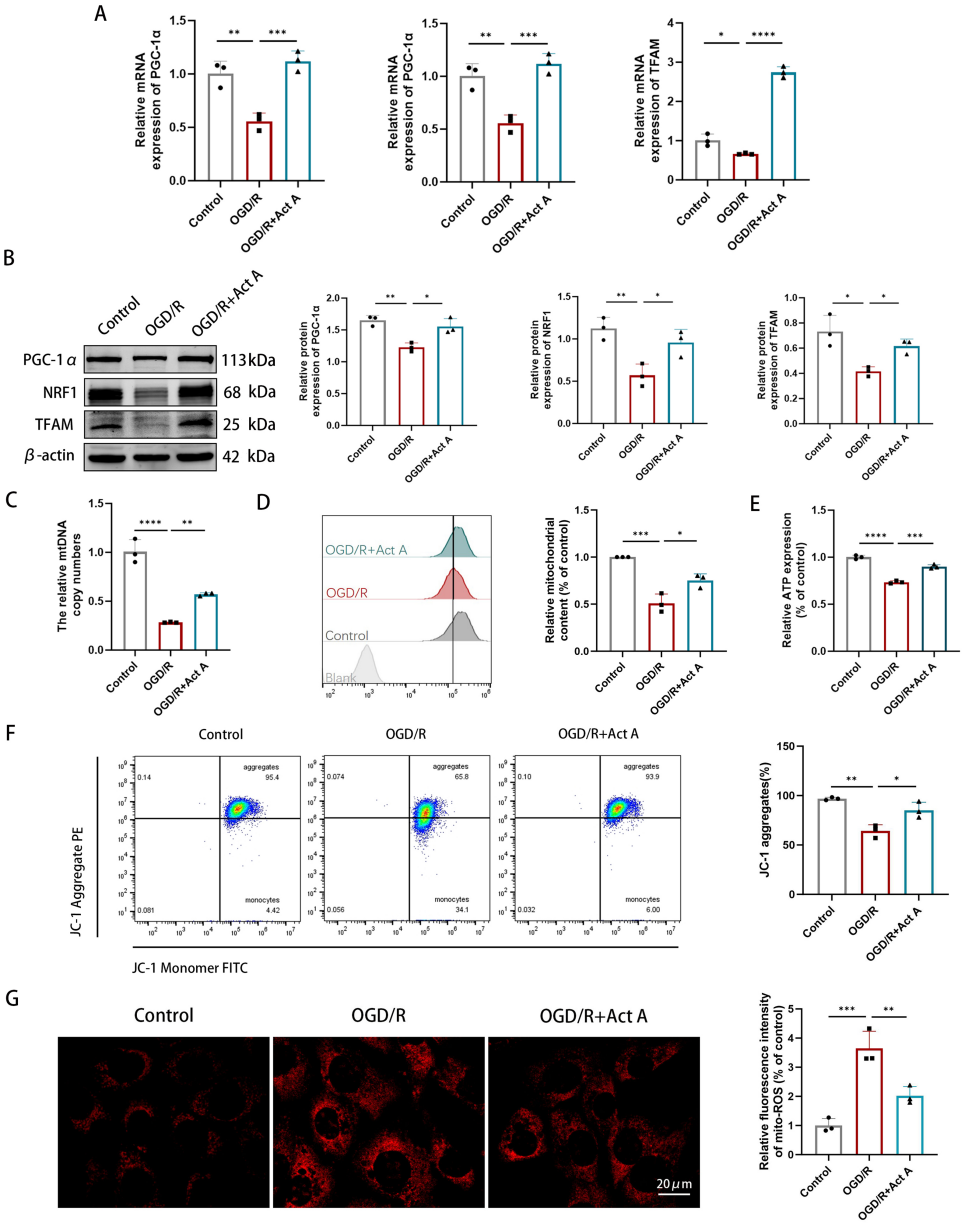
Act A alleviates mitochondrial dysfunction in OGD/R cells by activating the PGC-1α/NRF1/TFAM pathway to promote mitochondrial biogenesis. **(A)** qRT-PCR analysis of PGC-1α/NRF1/TFAM mRNA levels (*p < 0.05; **p < 0.01; ***p < 0.001; ****p < 0.0001, *n* = 3/group). **(B)** Western blot quantification of PGC-1α/NRF1/TFAM protein expression (*p < 0.05; **p < 0.01, *n* = 3/group). **(C)** Relative mtDNA copy number (ND1/β-actin ratio) measured by qRT-PCR (**p < 0.01; ****p < 0.0001, *n* = 3/group). **(D)** Mitochondrial mass assessed by MitoTracker™ Green staining and flow cytometry (*p < 0.05; ***p < 0.001, *n* = 3/group). **(E)** Cellular ATP levels detected by luciferase assay (*p < 0.05; **p < 0.01, *n* = 3/group). **(F)** Mitochondrial membrane potential (ΔΨm) measured by JC-1 staining and flow cytometry (*p < 0.05; **p < 0.01, *n* = 3/group). **(G)** Mitochondrial ROS levels evaluated by MitoSOX™ Red staining and confocal microscopy (scale bar, 20 μm, **p < 0.01; ***p < 0.001, *n* = 3/group). Data are represen ted as mean ± SEM.

Subsequently, we elucidated the regulatory role of Act A in mitochondrial biogenesis following I/R injury. qRT-PCR revealed that OGD/R suppressed the transcription of mitochondrial biogenesis pathway-related genes, including PGC-1α, NRF1, and TFAM. Act A administration significantly elevated mRNA expression levels of these genes compared to the OGD/R group, suggesting the transcriptional activation of mitochondrial biogenesis (Figure 3A). Western blotting confirmed congruent upregulation of PGC-1α, NRF1, and TFAM (Figure 3B). Quantification of the relative copy number of mtDNA by qRT-PCR indicated that treatment with Act A significantly increased the ND1/*β*-actin ratio in OGD/R cells (Figure 3C). Mitochondrial mass was assessed using MitoTracker™ Green staining coupled with flow cytometry. OGD/R cells exhibited significantly reduced mitochondrial fluorescence intensity compared to control cells, while treatment with Act A markedly restored fluorescence intensity (Figure 3D). Collectively, these findings suggest that Act A restored mitochondrial biogenesis.

Luciferase assay showed significantly decreased ATP levels in OGD/R cells compared to control cells, which was reverted after treatment with Act A (Figure 3E). JC-1 staining revealed significantly increased green fluorescence (depolarized mitochondria) in OGD/R cells, whereas Act A restored the red/green fluorescence ratio, suggesting the stabilization of mitochondrial membrane potential (Figure 3F). MitoSOX™ Red staining confirmed significantly elevated levels of mitochondrial ROS in OGD/R cells, which was reversed by Act A (Figure 3G). These findings highlight that Act A effectively ameliorated OGD/R-induced impairment of mitochondrial energy metabolism, stabilized membrane potential, and attenuated mitochondrial dysfunction.

### 3.4 Activin A activated PGC-1α through canonical and non-canonical pathways

**Figure 4:**
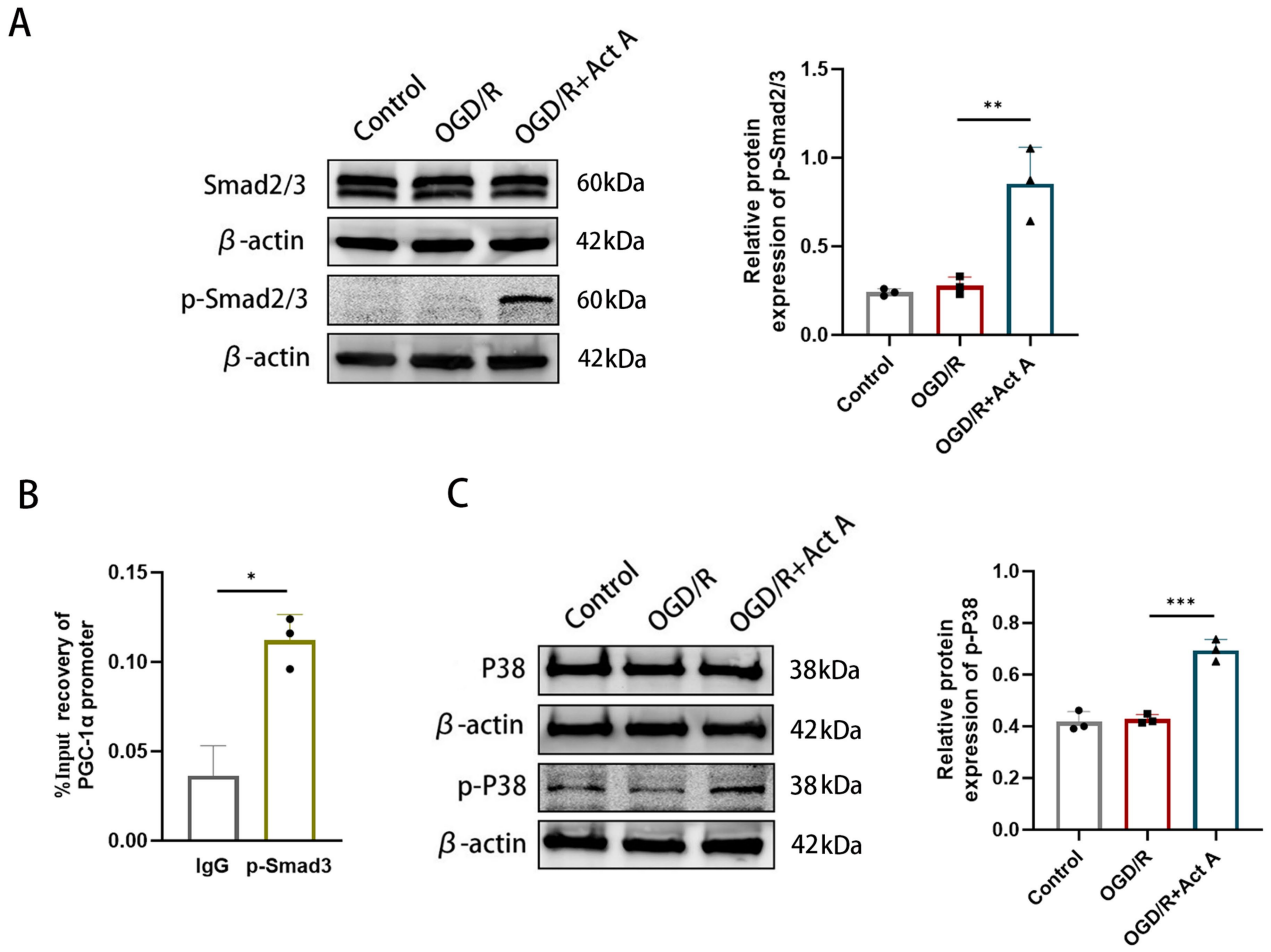
Act A activates PGC-1α through canonical and non-canonical signaling pathways. **(A)** Western blot analysis of phospho-Smad2/3 protein expression (**p < 0.01, *n* = 3/group). **(B)** Chromatin immunoprecipitation coupled with qPCR demonstrating physical interaction between p-Smad3 and the PGC-1α promoter (*p < 0.05, *n* = 3/group). **(C)** Western blot detection of phospho-p38 MAPK levels (***p < 0.001, *n* = 3/group). Data are represented as mean ± SEM.

To elucidate the molecular mechanisms underlying Act A-induced PGC-1α activation, we investigated both Smads and p38-MAPK signaling pathways downstream of Act A. As Act A primarily signals through the Smad family of transcription factors, we first assessed whether Act A activates PGC-1α via Smad signaling. Western blotting revealed low levels of p-Smad2/3 in both the control and OGD/R groups, whereas treatment with Act A significantly increased p-Smad2/3 expression compared to OGD/R alone (Figure 4A), suggesting Smad pathway activation. Since Smad3 functions as a transcriptional regulator, we conducted ChIP coupled with qPCR to determine whether p-Smad3 directly binds to the promoter region of the *PGC-1α* gene. Using three primer sets targeting the proximal promoter region, qPCR of p-Smad3-immunoprecipitated DNA demonstrated significantly enriched *PGC-1α* promoter fragments compared to negative controls (Figure 4B). This finding confirms the direct binding of p-Smad3 to the promoter region of the *PGC-1α* gene, suggesting that Act A transcriptionally regulates PGC-1α through Smad3 phosphorylation. Previous studies have indicated that phosphorylated p38-MAPK can activate PGC-1α-mediated mitochondrial biogenesis (23). Western blotting showed that treatment with Act A significantly upregulated phospho-p38 MAPK levels in the OGD/R+Act A group compared to the OGD/R group (Figure 4C), suggesting parallel activation of the p38-MAPK cascade.

### 3.5 PGC-1α silencing abrogated act A-mediated neuroprotection in cerebral I/R injury

**Figure 5:**
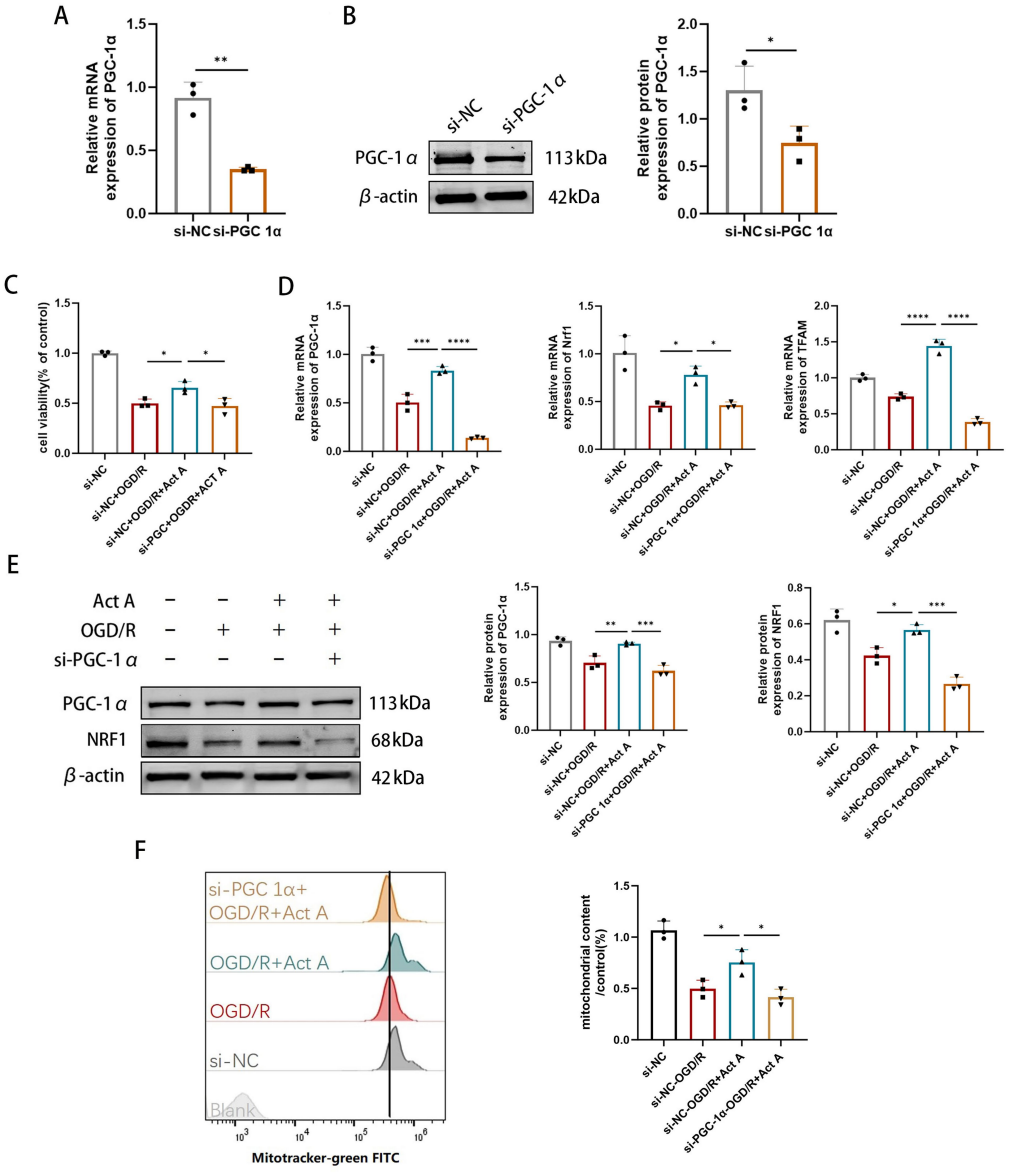
Silencing PGC-1α abolishes Act A-mediated mitochondrial biogenesis. **(A,B)** PGC-1α knockdown efficiency assessed by qPCR and western blot (*p < 0.05, **p < 0.01, *n* = 3/group). **(C)** *CCK-8 assay demon*strating the impact of *si-PGC-1α*-treated OGD/R cells with Act A intervention (*p < 0.05, *n* = 3/group). **(D,E)** qPCR and estern blot analysis of PGC-1α/NRF1/TFAM mRNA and protein expression alterations (*p < 0.05, **p < 0.01, ***p < 0.001, ****p < 0.0001, *n* = 3/group). **(F)** Flow cytometric quantification of mitochondrial mass (*p < 0.05, *n* = 3/group). Data are represented as mean ± SEM.

To determine whether Act A exerts neuroprotective effects against cerebral I/R injury by activating PGC-1α, we conducted siRNA-mediated PGC-1α silencing followed by phenotypic and functional analyses. qRT-PCR and Western blotting confirmed efficient PGC-1α knockdown, with the si-PGC-1α group exhibiting a 70% reduction in the mRNA expression levels of *PGC-1α* compared to si-NC controls (Figure 5A) and significantly diminished protein levels of PGC-1α (Figure 5B). We subsequently divided cells into four groups, including si-NC, si-NC+OGD/R, si-NC+OGD/R+Act A, and si-PGC-1α+OGD/R+Act A. CCK-8 viability assays demonstrated significantly reduced survival of cells in the si-PGC-1α+OGD/R+Act A group compared to the si-NC+OGD/R+Act A group (Figure 5C), confirming that the genetic ablation of PGC-1α abolished the neuroprotective effects of Act Aagainst OGD/R injury.

qRT-PCR and Western blotting of genes linked to the mitochondrial biogenesis pathway revealed significantly elevated mRNA and protein levels of PGC-1α, NRF1, and TFAM in si-NC+OGD/R+Act A cells compared to si-NC+OGD/R controls. This effect was abrogated in the si-PGC-1α+OGD/R+Act A group, demonstrating markedly reduced expression compared to the si-NC+OGD/R+Act A group (Figures 5D,E). These findings suggest that PGC-1α silencing blocked Act A-mediated upregulation of mitochondrial biogenesis machinery. Functional validation via MitoTracker™ Green staining coupled with flow cytometry showed significantly enhanced mitochondrial fluorescence intensity in cells in the si-NC+OGD/R+Act A group compared to the si-NC+OGD/R group. This effect was abolished in the si-PGC-1α+OGD/R+Act A group (Figure 5F), collectively confirming that the positive effects of Act A on mitochondrial biogenesis in cells subjected to OGD/R relied strictly on PGC-1α.

### 3.6 Silencing PGC-1α abolished act A-mediated amelioration of mitochondrial dysfunction and neuronal ferroptosis

**Figure 6:**
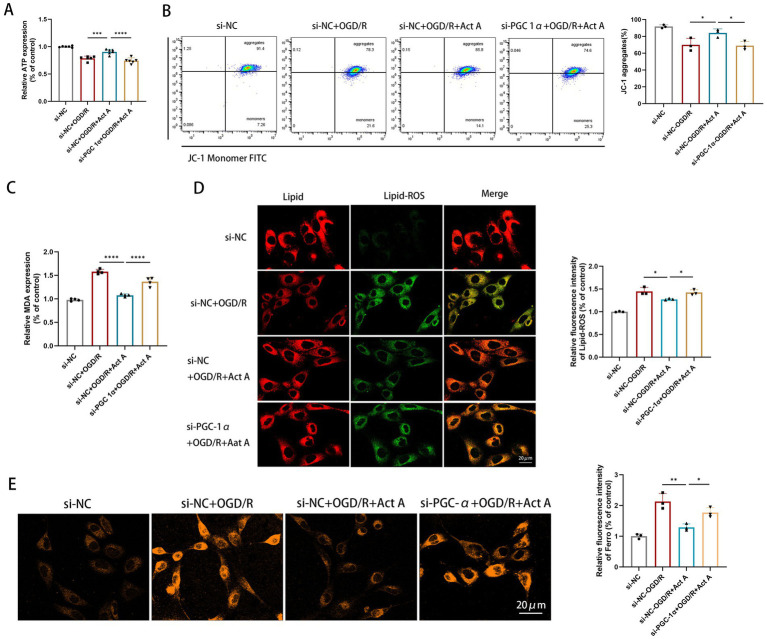
Silencing PGC-1α reverses activin A-mediated neuroprotection. **(A)** ATP levels in si-PGC-1α-treated OGD/R cells with Act A intervention (**p < 0.001, ***p < 0.0001; *n* = 3/group). **(B)** Mitochondrial membrane potential assessed by JC-1 staining and flow cytometry (*p < 0.05, *n* = 3/group). **(C)** *MDA con*tent quantification (**p < 0.001, ***p < 0.0001; *n* = 3/group). **(D,E)** Con focal microscopy analysis of lipid ROS and Fe^2+^ levels (scale bar, 20 μm, *p < 0.05; **p < 0.001, *n* = 3/group). Data are represented as mean ± SEM.

Intracellular ATP levels were quantified to determine the effect of PGC-1α knockdown on mitochondrial bioenergetics and membrane potential. The si-PGC-1α+OGD/R+Act A group exhibited significantly reduced ATP levels compared to the si-NC+OGD/R+Act A group (Figure 6A), suggesting that PGC-1α silencing reversed the beneficial effects of Act A on mitochondrial energy metabolism in OGD/R cells. JC-1 fluorescence probing revealed that mitochondrial membrane potential in the si-PGC-1α+OGD/R+Act A group was comparable to that in the si-NC+OGD/R and significantly diminished compared to si-NC+OGD/R+Act A (Figure 6B), demonstrating the reversal of the stabilizing effects of Act A on mitochondrial membrane potential.

Lipid peroxidation analysis showed significantly elevated levels of MDA in si-PGC-1α+OGD/R+Act A cells compared to si-NC+OGD/R+Act A cells (Figure 6C). Confocal imaging with C11-BODIPY probe and Ferro probe confirmed increased lipid ROS and Fe^2+^ levels in the si-PGC-1α+OGD/R+Act A group compared to the si-NC+OGD/R+Act A group (Figures 6D,E), suggesting that PGC-1α silencing abolished the inhibitory effects of Act A on lipid ROS and iron accumulation. Collectively, these results suggest that the protective effects of Act A against OGD/R-induced mitochondrial dysfunction and ferroptosis highly rely on PGC-1α signaling.

## 4 Discussion

Cerebral I/R injury represents a major contributor to neurological deterioration in patients with ischemic stroke. This complex pathological process involves multiple cell death pathways that result in irreversible neuronal damage and neurological deficits. Currently, effective neuroprotective agents remain clinically unavailable. Act A, a ~ 25 kDa secretory cytokine, functions as a pivotal neuromodulator in the central nervous system. This study aimed to determine whether Act A confers neuroprotection against cerebral I/R injury through PGC-1α-dependent mitochondrial biogenesis and elucidate the underlying signaling mechanisms. In the present study, treatment with Act A significantly attenuated cerebral I/R injury and neuronal ferroptosis while enhancing PGC-1α expression and mitochondrial biogenesis, thereby ameliorating mitochondrial dysfunction and oxidative stress. Mechanistically, Act A activated PGC-1α transcription through the canonical Smad pathway and promoted the post-translational activation of PGC-1α via the p38-MAPK cascade. This dual-pathway synergy enhanced mitochondrial biogenesis, restored mitochondrial function, and ultimately mediated the neuroprotective effects of Act A.

Both *in vivo* and *in vitro* models indicated that activin A significantly suppressed neuronal ferroptosis. Mechanistic experiments established phospholipid peroxides as the primary executors of ferroptosis. Within the lipid bilayer membrane, polyunsaturated fatty acids (PUFAs) anchor to phospholipids containing labile bis-allylic hydrogens that are readily abstracted by oxygen radicals, thereby generating lipid radicals. These lipid radicals subsequently form lipid peroxides through iron-catalyzed reactions with ROS, initiating a self-amplifying chain reaction (7, 24). Crucially, oxygen radicals are involved in both the initiation and propagation phases of this process. Mitochondria, as major cellular sources of ROS, play pivotal roles in the regulation of ferroptosis (25). Experimentally, mtDNA-depleted cells exhibited reduced susceptibility to erastin-induced ferroptosis (26). MitoQ, a mitochondria-targeted lipophilic antioxidant, significantly rescued GPX4 inactivation-mediated ferroptosis (25). Mitochondria possess two complementary anti-peroxidation systems, namely GPX4 and mitochondrial dihydroorotate dehydrogenase (DHODH), whose simultaneous inactivation induces pronounced mitochondrial lipid peroxidation and exacerbates ferroptosis (27, 28). Our previous study revealed that mitochondrial oxidative stress critically contributes to neuronal ferroptosis in cerebral I/R injury, while enhancing mitochondrial biogenesis was shown to effectively mitigate both mitochondrial oxidative stress and ferroptosis (13).

To determine whether the anti-ferroptotic effects of Act A rely on mitochondrial regulation, we investigated the effects of Act A on PGC-1*α* expression in both animal and cellular models. PGC-1α represents the master regulator of mitochondrial biogenesis, physiologically induced under energy-demanding conditions, such as fasting, cold exposure, and exercise. It stimulates the transcriptional activation of NRF1, NRF2, and estrogen-related receptor α (ERRα), subsequently upregulating TFAM to enhance the transcription and replication of mtDNA and promote mitochondrial biogenesis (29, 30). This study revealed elevated protein levels of PGC-1α in the cerebral cortex of Act A-treated rats compared to control rats merely subjected to I/R injury. In cellular studies, treatment with Act A significantly upregulated both the mRNA and protein expression levels of PGC-1α, NRF1, and TFAM compared to the OGD/R group, suggesting that treatment with activin A activated the mitochondrial biogenesis program through enhanced PGC-1α signaling.

Mitochondrial biogenesis constitutively proceeds in healthy cells to regulate mitochondrial abundance, morphology, and functionality, thereby meeting cellular energy, metabolic, and signaling demands (31). Aged cells typically exhibit diminished mitochondrial mass and reduced mitochondrial protein content, with attenuated biogenic capacity being correlated with decreased levels of PGC-1α (32). Aligned with prior findings, our study detected reduced neuronal expression of PGC-1α following cerebral I/R injury (33), concomitant with decreased mtDNA copy numbers and diminished mitochondrial quantity. These observations indicate compromised mitochondrial biogenesis, culminating in neuronal mitochondrial dysfunction. Notably, treatment with Act A robustly enhanced mitochondrial biogenesis, restored mitochondrial membrane potential, and potentiated cellular ATP production.

This study revealed that Act A synergistically coordinates PGC-1α expression and function through dual signaling pathways. Canonically, Act A binds to serine/threonine kinase receptors, leading to ActRI-mediated phosphorylation of Smad2/3. The activated Smad complex translocates to the nucleus and modulates the transcription of target genes (14). Our data indicated that treatment with Act A significantly upregulated phosphorylated Smad2/3 levels in OGD/R models, with chromatin immunoprecipitation confirming p-Smad3 binding to the promoter region of the *PGC-1α* gene, directly facilitating its transcriptional regulation. Concurrently, Act A substantially upregulated phosphorylated p38-MAPK. Activated p38-MAPK enhanced PGC-1α function through two distinct mechanisms: (1) relieving p160 myb-binding protein (p160 MBP)-mediated suppression of PGC-1α (34), and (2) phosphorylating the transcription factor cAMP response element-binding protein (CREB), which subsequently activates the transcription function of PGC-1α (35, 36). These findings establish PGC-1α as a critical downstream effector of Act A signaling. We also conducted PGC-1α silencing to definitively determine whether PGC-1α mediates the neuroprotective effects of Act A in cerebral I/R injury. Strikingly, treatment with Act A failed to rescue mitochondrial biogenesis or attenuate neuronal ferroptosis in silenced models, confirming the essential role of PGC-1α in this protective cascade.

In the central nervous system, Act A released from pyramidal neurons modulates microglial populations, shapes cortical circuitry, and stimulates oligodendrocyte precursor cell differentiation to promote myelination (37, 38). Notably, Act A is induced during autoimmune neuroinflammation, where it restricts the pathogenicity of Th17 cells and amplifies the expression of anti-inflammatory genes (39, 40). Following cerebral ischemia, Act A release surges rapidly upon transient hypoxia-ischemia, activating Smad signaling to prevent neuronal death (41). Previous studies have indicated that Act A confers neuroprotection against cerebral I/R injury by enhancing oligodendrocyte-mediated myelination, suppressing cGAS-STING-driven autophagy, and hindering neuronal ferroptosis through the NRF2 pathway (42–44). This study further delineated the neuroprotective effects of Act A against cerebral I/R injury through dual-pathway activation of PGC-1α. Functioning as the master regulator of nuclear-encoded mitochondrial genes, PGC-1α governs neuronal metabolism and differentiation, cortical function, and aging (45, 46). Treatment with Act A activated the PGC-1α/NRF1/TFAM signaling axis, restored mtDNA synthesis, increased mitochondrial biogenesis, and enhanced respiratory function. PGC-1α modulates cellular ferroptosis through multiple interconnected pathways. Primarily, it ameliorates mitochondrial oxidative stress, thereby downregulating the generation of pro-ferroptotic oxygen radicals (13). Additionally, PGC-1α suppresses the biosynthesis of PUFA, consequently limiting substrate availability for ferroptosis (47). Moreover, PGC-1α recruits nuclear factor NRF2 to the promoter region of the *GPX4* gene, where it functions as a transcriptional co-activator, ultimately inhibiting ferroptotic lipid peroxidation cascades (48).

Collectively, this study systematically indicated that Act A cooperatively activates PGC-1α through Smad3/p38-MAPK dual signaling pathways, ameliorating cerebral I/R injury by enhancing mitochondrial biogenesis and suppressing neuronal ferroptosis. These findings expand the molecular framework behind the neuroprotective properties of Act A and introduce novel therapeutically exploitable targets for reperfusion therapy after ischemic stroke. Through coordinated preclinical and clinical studies, future studies should establish optimal administration routes and pharmacokinetic profiles of exogenous Act A in patients with stroke to accelerate clinical translation. A critical hurdle remains the limited central nervous system delivery efficiency of macromolecular therapeutic proteins, necessitating combinatorial approaches with innovative delivery strategies to enhance blood–brain barrier penetration and ischemic lesion targeting. Concurrent development of stable, high-activity Act A recombinant formulations or potent small-molecule mimetics represents a pivotal direction for advancing clinical applications.

## Data Availability

The raw data supporting the conclusions of this article will be made available by the authors, without undue reservation.
